# Low Cardiac Output Leads Hepatic Fibrosis in Right Heart Failure Model Rats

**DOI:** 10.1371/journal.pone.0148666

**Published:** 2016-02-10

**Authors:** Yoshitaka Fujimoto, Takashi Urashima, Daisuke Shimura, Reiji Ito, Sadataka Kawachi, Ichige Kajimura, Toru Akaike, Yoichiro Kusakari, Masako Fujiwara, Kiyoshi Ogawa, Nobuhito Goda, Hiroyuki Ida, Susumu Minamisawa

**Affiliations:** 1 Department of Cell Physiology, The Jikei University School of Medicine, Tokyo, Japan; 2 Department of Pediatrics, The Jikei University School of Medicine, Tokyo, Japan; 3 Division of Cardiology, Saitama Children’s Medical Center, Saitama, Japan; 4 Department of Life Science and Medical Bioscience, Waseda University, Tokyo, Japan; Rutgers New Jersey Medical School, UNITED STATES

## Abstract

**Background:**

Hepatic fibrosis progresses with right heart failure, and becomes cardiac cirrhosis in a severe case. Although its causal factor still remains unclear. Here we evaluated the progression of hepatic fibrosis using a pulmonary artery banding (PAB)-induced right heart failure model and investigated whether cardiac output (CO) is responsible for the progression of hepatic fibrosis.

**Methods and Results:**

Five-week-old Sprague-Dawley rats divided into the PAB and sham-operated control groups. After 4 weeks from operation, we measured CO by echocardiography, and hepatic fibrosis ratio by pathological examination using a color analyzer. In the PAB group, CO was significantly lower by 48% than that in the control group (78.2±27.6 and 150.1±31.2 ml/min, P<0.01). Hepatic fibrosis ratio and serum hyaluronic acid, an index of hepatic fibrosis, were significantly increased in the PAB group than those in the control group (7.8±1.7 and 1.0±0.2%, P<0.01, 76.2±27.5 and 32.7±7.5 ng/ml, P<0.01). Notably, the degree of hepatic fibrosis significantly correlated a decrease in CO. Immunohistological analysis revealed that hepatic stellate cells were markedly activated in hypoxic areas, and HIF-1α positive hepatic cells were increased in the PAB group. Furthermore, by real-time PCR analyses, transcripts of profibrotic and fibrotic factors (TGF-β1, CTGF, procollargen I, procollargen III, MMP 2, MMP 9, TIMP 1, TIMP 2) were significantly increased in the PAB group. In addition, western blot analyses revealed that the protein level of HIF-1α was significantly increased in the PAB group than that in the control group (2.31±0.84 and 1.0±0.18 arbitrary units, P<0.05).

**Conclusions:**

Our study demonstrated that low CO and tissue hypoxia were responsible for hepatic fibrosis in right failure heart model rats.

## Introduction

Liver dysfunction is a frequent complication of heart failure, and also induces cardiac dysfunction which is known as cardiohepatic syndrome [1]. In the CHARM trial, increases in alanine aminotransferase (ALT) and total bilirubin (TB), and a decrease in albumin (Alb) were noted in 3%, 13%, and 18% of 2,679 patients with chronic heart failure, respectively [2]. Right heart failure-associated liver dysfunction is referred to as cardiac or congestive hepatopathy (CH).

CH was initially reported as ‘nutmeg liver’ by Sherlock et al. in 1951 [3]. CH progresses in stages with sinusoidal dilatation that results from a persistent elevation in hepatic venous pressure. In addition, oxygen/nutrient supply to hepatocytes decreases in CH as liver blood flow decreases, resulting in hepatocellular necrosis [4]. Accordingly, the main pathological findings of CH are sinusoidal dilatation and centrilobular necrosis [5]. Hepatic fibrosis is often found in CH, and in more severe conditions, CH may progress to cardiac cirrhosis [3]. Only a few studies have examined the progression of hepatic fibrosis in CH [6–8]. Myers et al. reported that hepatic fibrosis was found in 74% of 83 CH patients, and that there was no relationship between hepatic fibrosis and an increase in hepatic venous pressure [6]. The problem of clinical studies is that patient backgrounds (including disease and medication histories as well as age) are markedly biased. In turn, CH has not yet been extensively investigated using an animal heart failure model because of the lack of a good model. Thus, the causal factor of heart failure-induced hepatic fibrosis still remains unclear.

Although the mechanism of hepatic fibrosis progression has not yet been examined in detail, it is known that stellate cells play a primary role in hepatic fibrosis [9]. The role of stellate cells underlying hepatic fibrosis is as follows: liver dysfunction caused by congestion induces hepatocellular necrosis, and Kupffer cells that mobilize in the necrotic area, are activated, and release transforming growth factor-β1 (TGF-β1). Stellate cells are then activated by TGF-β1, and transform α-smooth muscle actin (α-SMA)-positive myofibroblasts. Activated myofibroblasts produce collagen and other extracellular matrices, resulting in fibrosis [10].

According to recent studies, tissue hypoxia is known to be one of the factors of hepatic fibrosis [11–13]. Hypoxia-inducible factor-1α (HIF-1α), which functions as a key transcription factor in response to hypoxia associates with the development of hepatic fibrosis [12]. Zhan L, et al. also reported that HIF-1α has potential as a novel therapeutic target for hepatic fibrosis [13]. Importantly, low CO causes tissue hypoxia in all organs including the liver [14]. Therefore, we hypothesized that low CO-induced hypoxia is responsible for hepatic fibrosis.

In this study, we have established a right heart failure model induced by PAB, and we evaluated hepatic fibrosis by pathological examination, and CO by echocardiography. This study is the first report of evaluated the relationship between CO and hepatic fibrosis in a heart failure model rat.

## Materials and Methods

### Experimental designs

**Fig 1:**
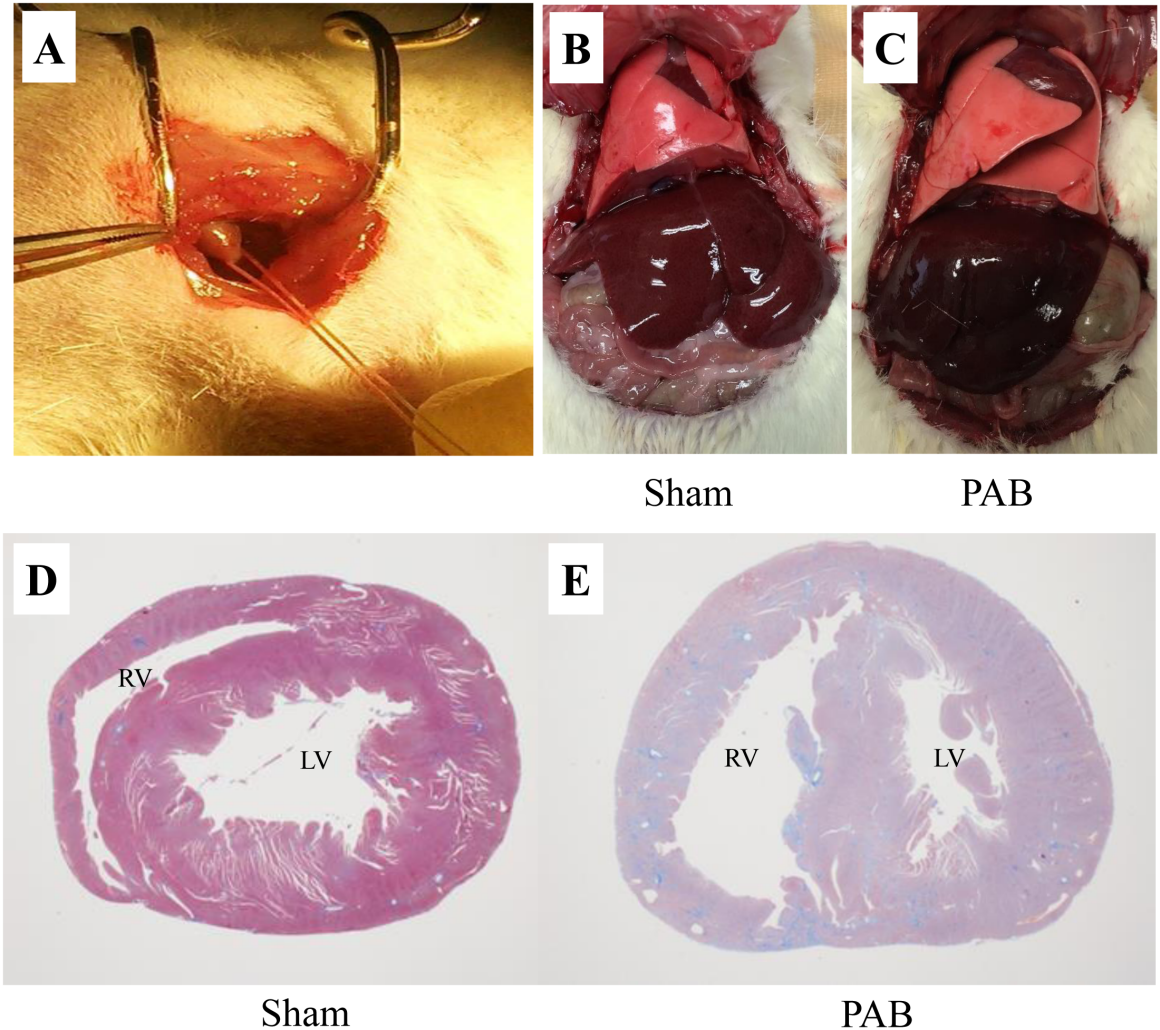
PAB procedure and pathological characteristic of the PAB liver and heart. Image (A): PAB was created by surrounding a tread at the main pulmonary artery. Image (B): The liver of sham-operated control. Image (C): The liver of PAB rat like nutmeg with darkish color. Image (D): The heart of the sham-operated control. Image (E): Hypertrophied right ventricular wall in the PAB heart.

Five-week-old male Sprague-Dawley rats (body weight: 150–250 g) were randomly divided into the PAB and sham-operated control groups. After intubation using Angiocath^™^18G for rats in the PAB group, respiration was artificially managed under anesthesia with 2% isoflurane using a Harvard rodent ventilator (Harvard Apparatus, Holliston, MA), setting the respiratory rate at 120-140/min and tidal volume at 10 μl/g. After thoracotomy through the left 4th intercostal region, the thymus was removed and the main pulmonary artery was ligated twice with the outer tube of BD Angiocath^™^20G (diameter, 1.88 mm) using 4.0 silk thread (Fig 1A). The outer tube was then removed after double ligature and the thorax was closed. In the sham-operated control group, thoracotomy was performed under artificial respiratory management, followed by thymectomy only, and the thorax was then closed.

The progression of hepatic fibrosis was evaluated in the model rats (the PAB group, n = 12, the sham-operated control group, n = 10). Echocardiography and blood sampling were performed 4 weeks after surgery, and tissue was collected after pentobarbital-induced euthanasia for pathological examination, immunostaining, and real-time PCR analyses.

All rats were maintained at 22±2°C under a 12-hour lighting cycle following the National Institute of Health guidelines for animal experiments. Experiments were performed after obtaining approval from the Animal Experiment Committee of The Jikei University School of Medicine.

### Echocardiography

**Fig 2:**
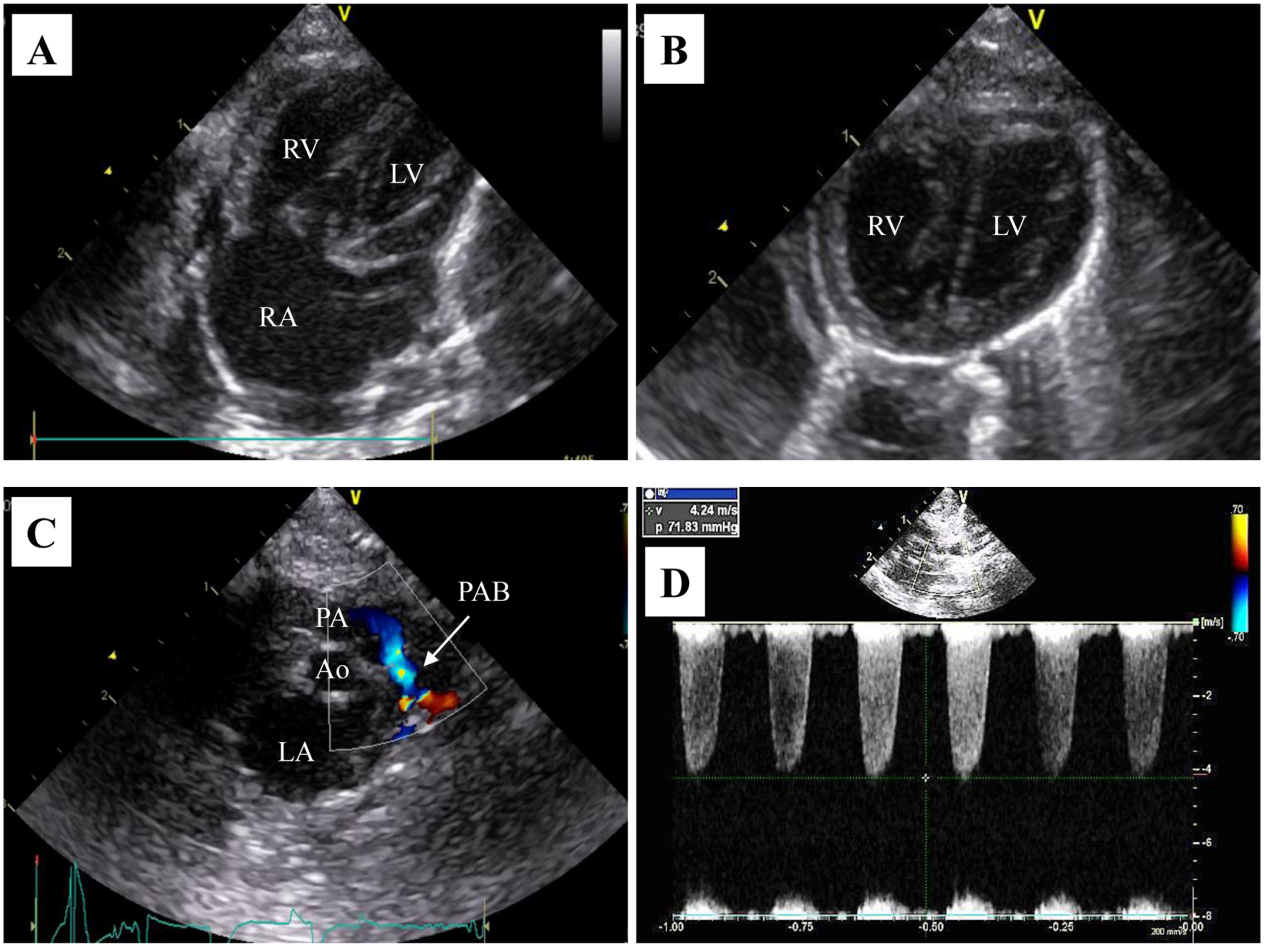
Echocardiographic characteristic of PAB rats. Image (A): The right-sided heart is enlarged in a PAB rat (4 chamber view). Image (B): The right ventricular wall and ventricular septum are hypertrophied in a PAB rat (short axis view). Image (C): A color Doppler image of the blood flow of the pulmonary artery before and after the pulmonary artery banding region (short axis view). Image (D): The pressure gradient between the pulmonary artery and RV was calculated from the blood flow velocity in the PAB region using Bernoulli’s principle (pressure gradient = 4 × v^2^).

Echocardiography was performed using the 12-MHz transducer (GE Vivid i8 ultra sound system, GE Healthcare, Milwaukee, WI). Measurements were performed using Echo Pac software (GE Healthcare), and the right ventricular (RV) outflow tract area (RVOT-area), RV outflow tract velocity time integral (RVOT-VTI), heart rate, blood flow velocity in the PAB region, and right atrial (RA) area were measured. The RV-pulmonary artery (PA) pressure gradient was calculated from the blood flow velocity in the PAB region using Bernoulli’s principle (pressure gradient = 4 × v^2^) (Fig 2D). We used RV-PA pressure gradient for the index of RV pressure. The RA dimension was measured from the four-chamber view using the area-length method as the index of central venous pressure. Cardiac output (CO) was calculated from the RVOT-area, RVOT-VTI, and heart rate using the following equation: CO (mL/min) = RVOT-VTI × HR × RVOT-area. For RVOT-VTI and heart rate, the mean of 3 heart beats was used. Fig 2 showed the echocardiographic characteristic of a PAB rat.

### Pathological examination and measurement of the fibrosis ratio

**Fig 3:**
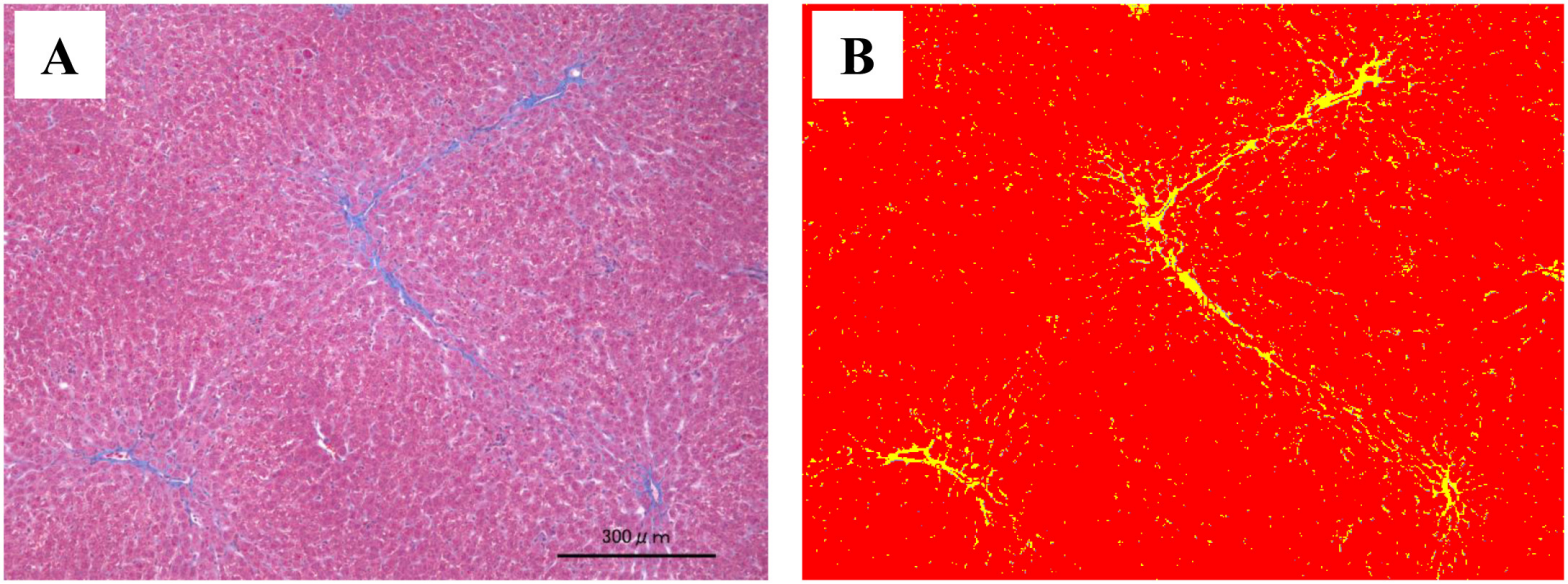
Measurement of the fibrotic zone using the Image-Pro^®^Premier 9.1. Image (A): Liver stained using Masson-Trichrome stain. (Original magnification, ×10). Image (B): Bicolor changes from Image (A) using Image-Pro^®^Premier 9.1. The percentage of fibrotic zone = yellow area / red area + yellow area.

**Fig 4:**
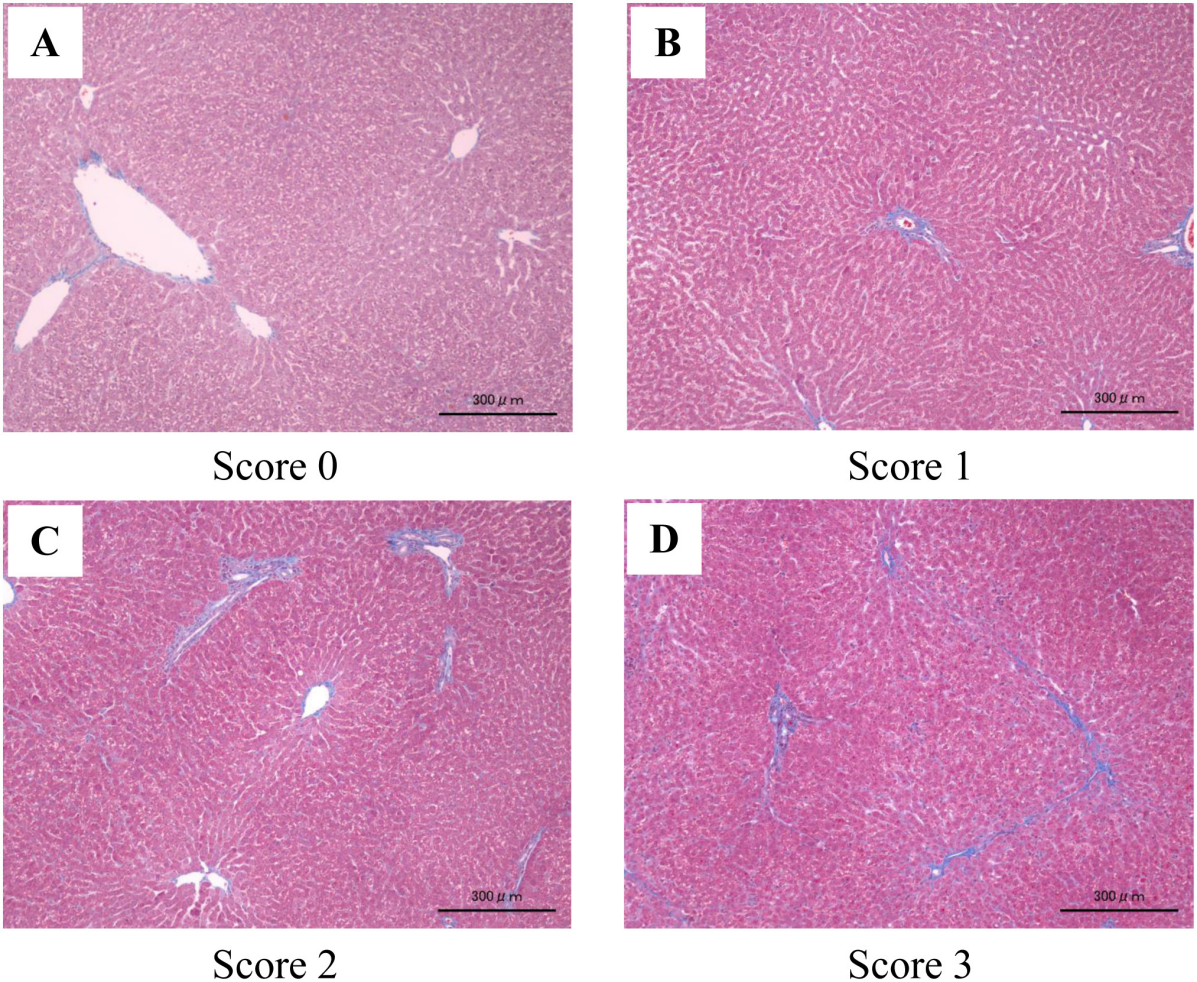
Congestive hepatic fibrosis score. Liver stained using Masson-Trichrome stain. (Original magnification, ×4). Image (A): score 0, absence of fibrosis. Image (B): score 1, central fibrosis. Image (C): score 2, central zone and portal fibrosis. Image (D): score 3, bridging fibrosis.

RA, RV, and liver specimens were excised and stained using Masson-trichrome stain. Slides of the excised specimens were observed at 10-times magnification under a light microscope. Six visual fields were randomly selected, in which fibrotic and other regions were converted to a 2-color gradation using Image-Pro^®^Premier 9.1 (Fig 3) (Media Cybernetics, Washington, WA). The fibrosis ratio was then measured from the fibrotic tissue area in each visual field, and the means of six visual fields were compared. To reduce visual field-related errors, visual fields containing no blood vessels were selected in the RA and RV, while those containing no portal vein-perfused regions in the liver were selected. To accurately evaluate hepatic fibrosis, the grade of fibrosis was evaluated in each preparation using the congestive hepatic fibrosis score [5]. This is a pathological classification method, in which a score of 0 represents the absence of fibrosis; 1, central fibrosis; 2, central zone and portal fibrosis; 3, bridging fibrosis. (Fig 4)

### Blood chemically examination

Aspartate aminotransferase (AST), ALT, TB, and alkaline phosphatase (ALP) were measured to evaluate liver function in the model rats. Hyaluronic acid was measured as an index of hepatic fibrosis. AST, ALT, TB and ALP were measured using a Hitachi 7180 (Hitachi, Japan), and hyaluronic acid was measured using a JCA-BM 8000 (Nihondenshi, Japan) fully automated clinical chemistry analyzer.

### Immunostaining

To detect tissue hypoxia, pimonidazole (60 mg/kg) was injected into the tail vein in the PAB (n = 4) and sham-operated control (n = 4) groups. Pathological specimens were collected on autopsy one hour after the injection, and were subjected to immunostaining using Hypoxyprobe^™^ (Cosmobio, Japan). In addition, immunohistological analysis of α-SMA and HIF-1α were performed in the same number of animals using mouse anti-human actin antibody (M0851, DakoCytomation, Denmark and NB100-105, Novus Biologicals, Canada, respectively).

### Real-time PCR analysis

TGF-β1, connective tissue growth factor (CTGF), procollargenI, procollargen III, matrix metalloproteinase 2 (MMP2), MMP9, tissue inhibitor of metalloproteinase 1 (TIMP1), TIMP2, HIF-1α and vascular endothelial growth factor (VEGF) mRNA expression levels in the liver were measured in the PAB (n = 4) and sham-operated control (n = 4) groups using real-time PCR. A liver fragment was immersed in 1 mL of Sepasol-RNA Super G (NACALAI TESQUE, Japan) and crushed using a bead-type homogenizer. After centrifugation at 12,000 g at 4°C for 15 minutes, the supernatant was collected and total RNA was extracted following the instructions attached to the kit. cDNA was synthesized using a TaKaRa PCR Thermal Cycler Dice ^™^ (TAKARA BIO, Japan), and RT-PCR was performed using a Thermal Cycler Dice ^®^ and SYBR ^®^ Premix Ex Taq^™^ (TAKARA BIO, Japan). The nucleotide sequences of the primers used are shown in Table 1. The 18S rRNA expression level was quantitated as an internal reference. Both isolation of total RNA from pooled tissues and generation of cDNA and reverse transcription-PCR analysis were performed as described previously [15].

**Table 1:** List of primer sequences used for real-time PCR.

Gene name	Forward primer	Reverse primer
**18S**	5'-AGCCTGAGAAACGGCTACC-3'	5'-TCCCAAGATCCAACTACGAG-3'
**TGF-β1**	5'-CTTTGTACAACAGCACCCGC-3'	5'-TAGATTGCGTTGTTGCGGTC-3'
**CTGF**	5'-CGGAGCGTGATCCCTGCGAC-3'	5'-GGTGCACCATCTTTGGCAGTGC-3'
**Procollargen I**	5'-GAGCGGAGAGTACTGGATCGA-3'	5'-CTGACCTGTCTCCATGTTGCA-3'
**Procollargen III**	5'-TGCCATTGCTGGAGTTGGA-3'	5'-GAAGACATGATCTCCTCAGTGTTGA-3'
**MMP2**	5'-TGGTGTGGCACCACCGAGGA-3'	5'-TCCACCCACAGTGGACATAGCAGT-3'
**MMP9**	5'-ACTCGAGCCGACGTCACTGT-3'	5'-GGCCCTCGCCGGTACAGGTA-3'
**TIMP1**	5'-TTCCGGTTCGCCTACACCCCA-3'	5'-TCCTTAAACGGCCCGCGATGA-3'
**TIMP2**	5'-TGTGCCCTGGGACACGCTTA-3'	5'-GCGTGTGATCTTGCACTCGCA-3'
**HIF-1α**	5'-TGCTCATCAGTTGCCACTTC-3'	5'-CATGGTCACATGGATGGGTA-3'
**VEGF**	5'-CCGGACGGGCCTCTGAAACC-3'	5'-GGTGCAGCCTGGGACCACTTG-3'

18S: 18S ribosomal RNA;

TGF-β1: transforming growth factor-β1;

CTGF: connective tissue growth factor;

MMP: Matrix metalloproteinase;

TIMP: Tissue inhibitor of Metalloproteinase;

HIF-1α: Hypoxia-inducible factor-1α;

VEGF: vascular endothelial growth factor.

### Western blot analysis

Total protein was extracted from the liver and used for Western blot analyses including antibodies against HIF-1α (NB100-123, Novus Biologicals, Canada) and β-actin (A5441, SIGMA, Japan), as described previously [15]. Briefly, tissues were homogenized in an ice-cold buffer [10 mM imidazole (pH 7.0), 300 mM sucrose, 5 mM sodium pyrophosphate decahydrate and 1 mM dithiothreitol with protease inhibitors (Complete Mini, Roche, Basel, Switzerland). To detect HIF1α and VEGF, we extracted proteins using cell lysis buffer (#9803, Cell signaling, Danvers, MA) with protease inhibitors (Complete Mini and 1 mM PMSF). Protein content was determined using the Bradford assay (Bio-Rad, Hercules, CA) and/or BCA methods (Thermo Fisher Scientific Inc., Waltham, MA), and bovine serum albumin (BSA) was used as a standard. The protein samples were separated using SDS-polyacrylamide gel electrophoresis or NuPAGE^®^ electrophoresis (Invitrogen, CA, USA) and transferred to polyvinylidene difluoride membranes (Millipore Corporation, Billerica, MA). When the molecular size of the target proteins was similar, we reused the same membrane for a different antibody after washing the membrane with a stripping buffer [62.5 mM Tris (pH 6.8), 100 mM 2-mercaptoethanol and 2% SDS]. After application of a secondary antibody, quantification of the target signals was performed using the LAS-3000 imaging system (Fujifilm, Tokyo, Japan) or EZ-Capture MG (ATTO, Tokyo, Japan).

### Statistical analysis

Results are presented as the mean ± standard error of the mean (SEM). The fibrosis ratio and serum hyaluronic acid level were compared between the PAB and sham-operated groups using the unpaired *t*-test, while various mRNA expression levels from real-time PCR were compared between these groups using the Mann-Whitney U-test. P<0.05 was considered significant.

## Results

### PAB rats developed right heart failure

Baseline characteristics of the PAB (n = 12) and sham-operated control (n = 10) groups are shown in Table 2. No significant differences were noted in body weight before surgery or on autopsy. The RV weight and RV weight/LV+IVS weight were significantly increased by approximately 3-fold in the PAB group than those in the sham-operated group (107±24.1 and 380±83.5 mg, 0.14±0.042 and 0.43±0.12 mg, P<0.01). Liver weight was not significantly different between the PAB and sham-operated groups.

**Table 2:** Echocardiographic and blood chemically examination parameters of the PAB and sham-operated control groups.

	Control	PAB	P value
**N**	10	12	
**BW operation (g)**	192.3±13.6	213.8±30.2	NS
**BW sacrifice (g)**	364.5±20.1	372.5±39.2	NS
**RV weight (mg)**	107±24.1	380±83.5	P<0.01
**RV weight/LV+IVS weight**	0.14±0.042	0.43±0.12	P<0.01
**Liver wight (g)**	14.6±1.49	14.6±2.25	NS
**Liver weight/BW (%)**	4.0±0.27	3.92±0.35	NS
**Echocardiography**			
**RV-PA pressure gradient (mmHg)**		68.9±19.7	
**RA dimension (cm**^**2**^**)**	0.25±0.058	0.63±0.14	P<0.01
**Cardiac output (ml/min)**	150.1±31.2	78.2±27.6	P<0.01
**Blood chemically examination**			
**AST (IU/L)**	149.9±52.4	184.4±52.7	NS
**ALT (IU/L)**	37.6±7.00	41.1±12.6	NS
**TB (mg/dl)**	0.036±0.0051	0.041±0.04	NS
**ALP (IU/L)**	1197±209.6	1229±414.0	NS
**Hyaluronic acid (ng/ml)**	32.7±7.51	76.2±27.5	P<0.01

Values are presented as the mean ± SD;

n: number of rats;

BW: body weight;

RV: right ventricular;

LV: left ventricular;

IVS: interventricular septum;

PA: pulmonary artery;

RA: right atrium;

AST: aspartate aminotransferase;

ALT: alanine aminotransferase;

TB: total bilirubin;

ALP: alkaline phosphatase;

PAB: pulmonary artery banding;

NS: not significant.

In the PAB group, CO measured by echocardiography was significantly lower by 48% than that in control group (78.2±27.6 and 150.1±31.2 ml/min, P<0.01). RA dimension was also significantly increased in the PAB group (150±31.2 and 78.2±27.6 cm^2^, P<0.01). RV-PA pressure gradient was increased (68.9±19.7 mmHg), and tricuspid regurgitation was moderate in all PAB rats.

### PAB rats developed hepatic fibrosis

Serum hyaluronic acid levels, an index of hepatic fibrosis, were significantly increased in the PAB group compared with those in the control group (76.2±27.5 and 32.7±7.5 ng/ml, P<0.01).

**Fig 5:**
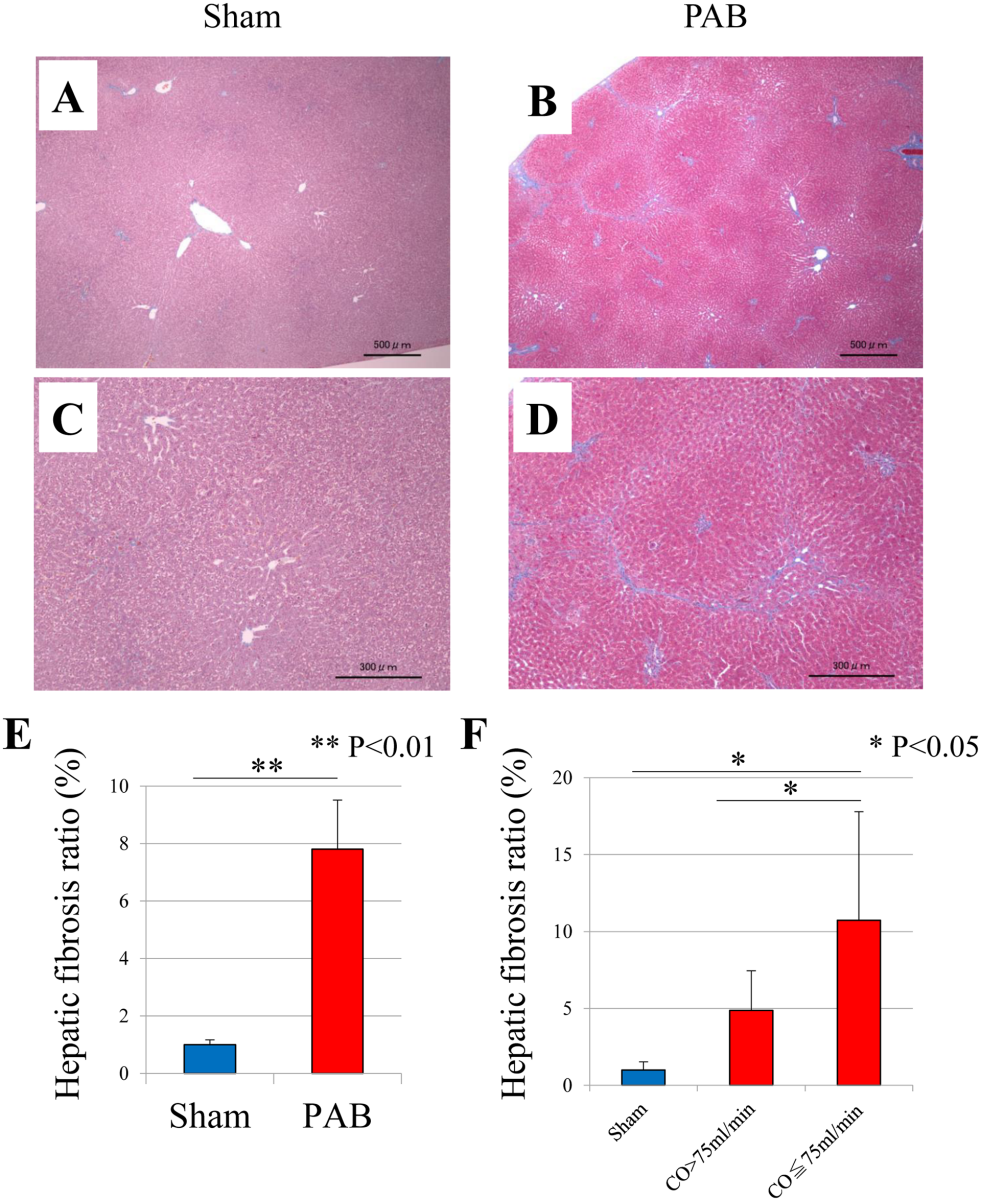
Pathological investigation and hepatic fibrosis ratio. (A) The liver of sham-operated controls stained using Masson-Trichrome stain (original magnification ×4). (B) The liver of PAB stained using Masson-Trichrome stain (original magnification ×4). (C) The liver of sham-operated controls stained using Masson-Trichrome stain (original magnification ×10). (D) The liver of PAB stained using Masson-Trichrome stain (original magnification ×10). (E) PAB rats (n = 12) compared with sham-operated controls (n = 10) for hepatic fibrosis ratio (mean ± SEM). (F) Divided the PAB group into two groups: PAB with preserved CO group (CO≥60 ml/min, n = 6) and PAB with reduced CO group (CO<60 ml/min, n = 6). Hepatic fibrosis ratio was significantly increased in PAB with reduced CO group (mean ± SEM). PAB: pulmonary artery banding.

Histological examination revealed that fibrosis was markedly promoted in the liver in the PAB group (Fig 5A–5D). Furthermore, fibrosis ratio using a color analyzer was significantly increased in the PAB group than that in the control group (7.8±1.7 and 1.0±0.2%, P<0.01) (Fig 5E).

We divided the PAB group into two groups: the PAB with preserved CO group (CO≥60 ml/min, n = 6) and the PAB with reduced CO group (CO<60 ml/min, n = 6). Hepatic fibrosis ratio was increased more in the PAB with reduced CO group (P<0.05).

In the sham-operated control group, the congestive hepatic fibrosis score was all score 0. Furthermore, the score of the PAB group was individual difference (Table 3). The score was highly correlated with the fibrosis ratio.

**Table 3:** Congestive hepatic fibrosis score of the PAB and sham-operated control groups.

Congestive hepatic fibrosis score	Control	PAB
**Score 0**	10	0
**Score 1**	0	4
**Score 2**	0	6
**Score 3**	0	2

PAB: pulmonary artery banding.

### The degree of hepatic fibrosis significantly correlated a decrease in CO

**Fig 6:**
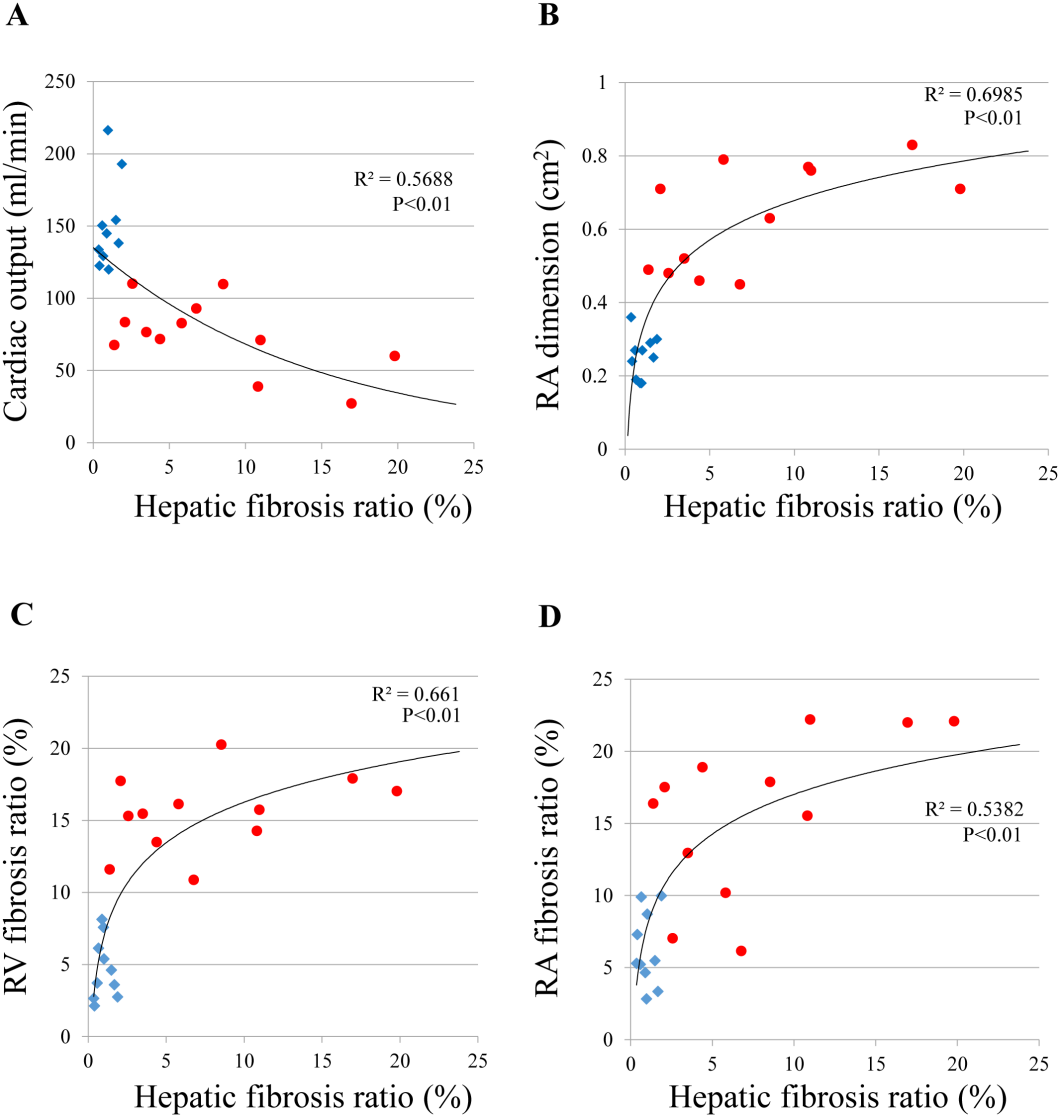
The correlation between hepatic fibrosis ratio and cardiac parameters. RA: right atrium; RV: right ventricular; PAB: pulmonary artery banding. Red circle plot: pulmonary artery banding model rat group (n = 12); blue square plot: sham-operated controls (n = 10). (A) Hepatic fibrosis progressed more in animals with a low CO. (B) Hepatic fibrosis progressed more in animals with an increased RA dimension. (C) Hepatic fibrosis progressed more in animals with an increased RV fibrosis. (D) Hepatic fibrosis progressed more in animals with an increased RV fibrosis.

The relationship between hepatic fibrosis and cardiac parameters is shown in Fig 6. Hepatic fibrosis progressed more in animals with a low CO and in those with an increased RA dimension (Fig 6A: R^2^ = 0.5688, P<0.01, 6B: R^2^ = 0.6985, P<0.01). Additionally, RA and RV fibrosis ratios were also increased with progression of hepatic fibrosis (Fig 6C: R^2^ = 0.661, P<0.01, 6D: R^2^ = 0.5382, P<0.01).

### Myofibroblasts were increased in the hypoxic area of the PAB liver

**Fig 7:**
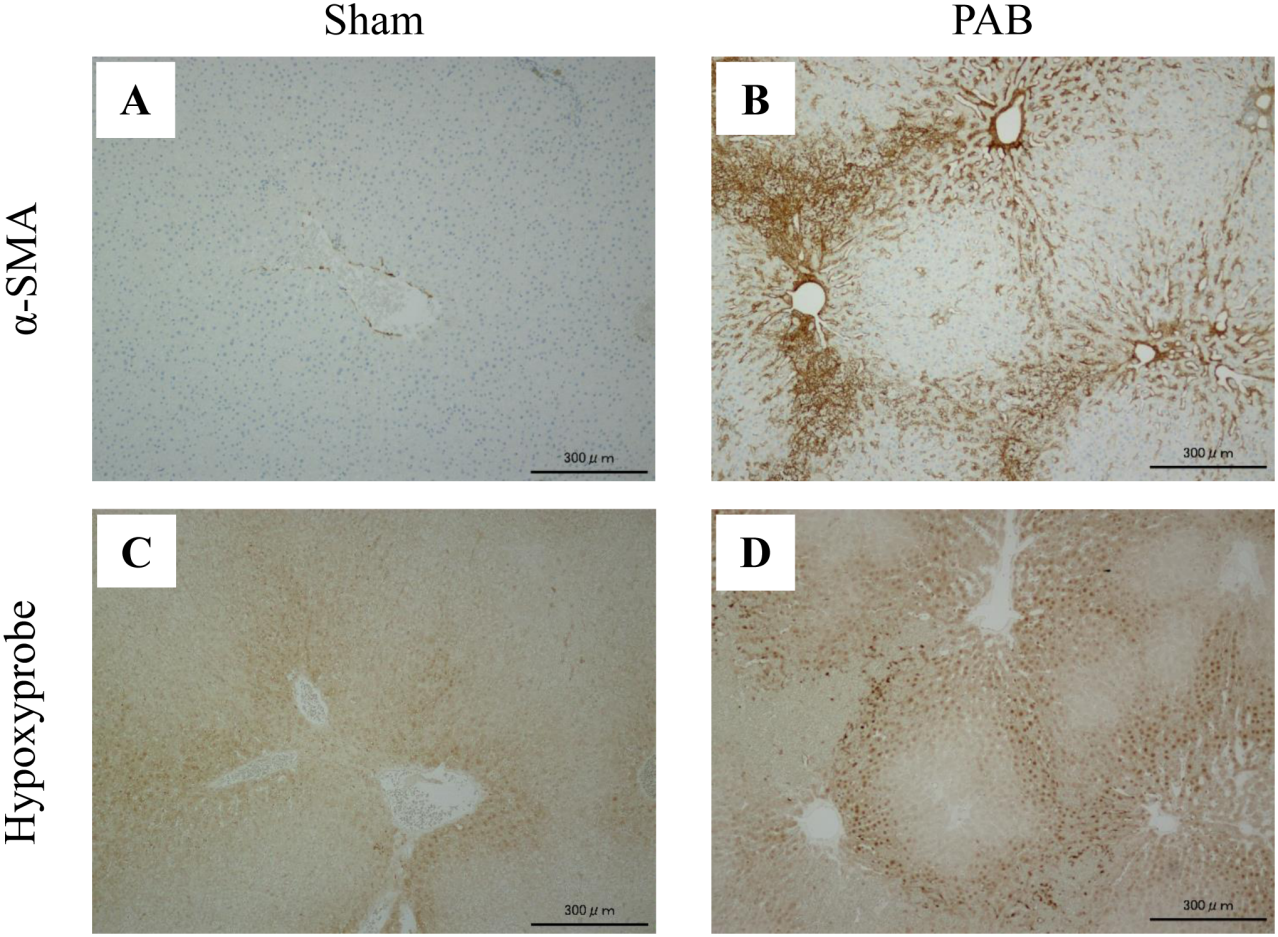
Immunostaining of the livers in the PAB and sham-operated control groups. PAB: pulmonary artery banding; α-SMA: alpha-smooth muscle actin. (Original magnification, ×10). (A) The sham-operated control liver stained by α-SMA. (B) The PAB liver stained by α-SMA. (C) The sham-operated control liver stained by Hypoxyprobe^™^. (D) The PAB liver stained by Hypoxyprobe^™^. Activated hepatic stellate cells were mostly observed in the hypoxic areas of the PAB liver.

α-SMA-positive myofibroblasts were increased in a pseudolobular pattern in PAB rat liver compared with controls (Fig 7A and 7B). Immunostaining using pimonidazole demonstrated that the hypoxic regions were significantly larger in the liver of PAB rats than in that of the sham-operated rats, and they were particularly prominent around the central vein (Fig 7C and 7D). Importantly, hepatic stellate cells were markedly activated in tissue hypoxic areas of the PAB liver.

### Transcripts of profibrotic and hypoxic factors were increased in PAB rats

**Fig 8:**
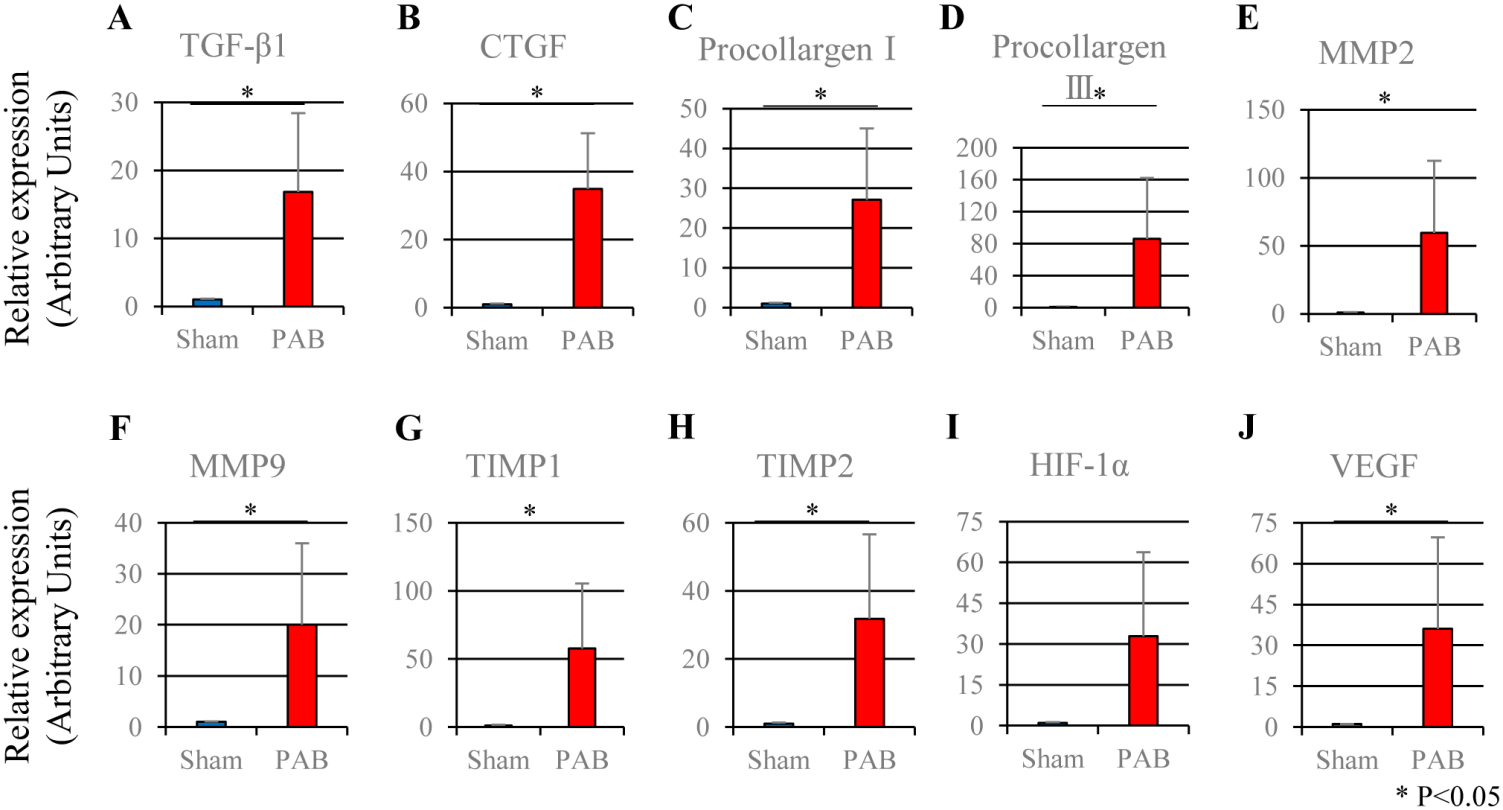
The mRNA expression levels in the liver of the PAB and sham-operated control groups. TGF-β1: transforming growth factor-β1; CTGF: connective tissue growth factor; MMP: matrix metalloproteinase; TIMP: tissue inhibitor of metalloproteinase; HIF-1α: hypoxia-inducible factor-1α; VEGF: vascular endothelial growth factor; PAB: pulmonary artery banding, (mean ± SEM), * P<0.05. (A) 34.9±16.4 and 1.0±0.15 arbitrary units, P<0.05 (B) 16.8±11.6 and 1.0±0.10 arbitrary units, P<0.05 (C) 27.1±18.0 and 1.0±0.22 arbitrary units, P<0.05 (D) 85.9±76.5 and 1.0±0.44 arbitrary units, P<0.05 (E) 59.4±53.1 and 1.0±0.23 arbitrary units, P<0.05 (F) 20.0±15.9 and 1.0±0.11 arbitrary units, P<0.05 (G) 57.5±47.8 and 1.0±0.43 arbitrary units, P<0.05 (H) 31.8±24.9 and 1.0±0.30 arbitrary units, P<0.05 (I) 32.8±31.0 and 1.0±0.33 arbitrary units (J) 36.0±33.6 and 1.0±0.07 arbitrary units, P<0.05.

The mRNA expression levels of profibrotic factors (TGF-β1, CTGF) and fibrotic factors (procollargen I, procollargen III) were significantly increased in the PAB group compared with those in the sham-operated control group (Fig 8A–8D). Furthermore, the mRNA expression levels of MMPs (MMP 2, MMP 9) were significantly increased in the PAB group (Fig 8E and 8F). TIMPs (TIMP 1, TIMP 2) were also increased in the PAB group (Fig 8G and 8H). In addition, the mRNA expression levels of hypoxic factors (HIF-1α, VEGF) were increased in the PAB group than those in the control group, although the increase in the HIF-1α mRNA expression did not reach a statistical significance (Fig 8I and 8J).

### The location and expression level of HIF-1α protein in the liver of PAB rats

**Fig 9:**
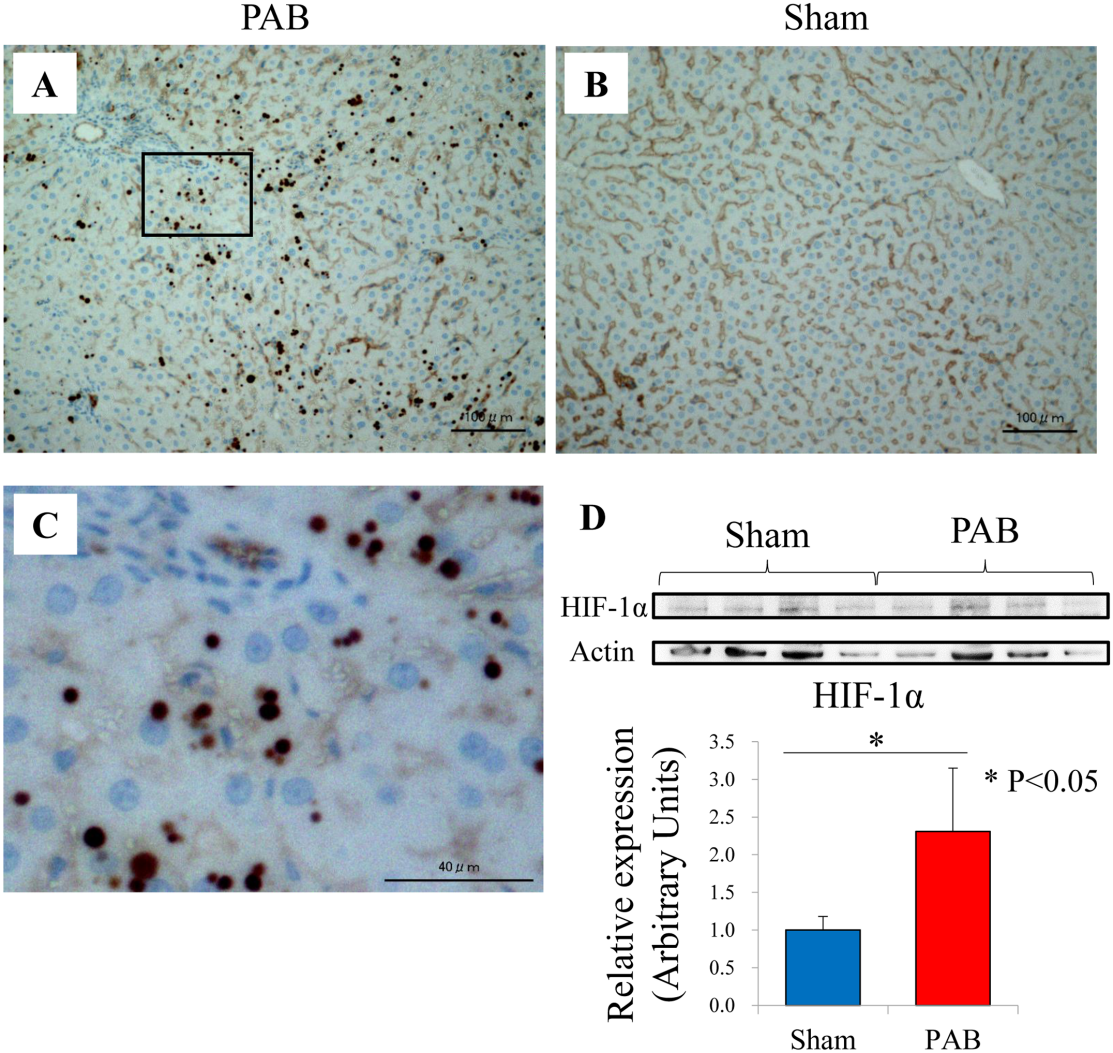
Immunohistological and western blot analyses of HIF-1α. (A) PAB liver stained by HIF-1α (original magnification, ×20). (B) Sham-operated control liver stained by HIF-1α (original magnification, ×20). (C) Enlarged view of the boxed area in the Image (A) (original magnification, ×100). (D) Western blot analysis of HIF-1α (mean ± SEM), * P<0.05. HIF-1α: hypoxia-inducible factor-1α; PAB: pulmonary artery banding.

The immunohistological analysis revealed that HIF-1α translocated into the nucleus in the liver of PAB rats (Fig 9A and 9C). Furthermore, western blot analysis revealed that the protein level of HIF-1α was significantly increased in the PAB group in that in the control group (2.31±0.84 and 1.0±0.18 arbitrary units, P<0.05, Fig 9D).

## Discussion

The most striking finding in the present study is that hepatic fibrosis in right heart failure progresses with a decrease in CO, and this low CO makes liver tissue relatively hypoxic. Hepatic fibrosis in CH has not been intensively investigated, because there was no good animal model of right heart failure to study CH. PAB stresses the RV though pressure overload to induce right heart failure for a short period. Impairment of RV function causes the elevation of central venous pressure. Therefore, PAB rats seem to be an excellent model to initially evaluate the mechanism of hepatic fibrosis due to right heart failure.

### Mechanism of hepatic fibrosis in CH

The symptom of CH is usually latent, because hepatic cells are not easily damaged by venous congestion itself. Along with this notice, there was no significant difference in AST, ALT, TB, and ALP between the two groups (Table 2), suggesting that hepatic cells are little damaged and there is no or little congestion of bile flow in the PAB liver. Therefore, the damage of hepatic cells is not likely accounted for the mechanism of CH. Instead, the increase in central venous pressure clinically contributes to hepatic fibrosis has been reported. Simonetto et al. recently reported that venous congestion-induced sinusoidal thrombosis using a mouse model with inferior vena cava stenosis [8]. In the present study, we observed apparent sinusoidal dilatation, but not sinusoidal thrombosis in our PAB rats, although hepatic fibrosis progressed in all of the PAB rats. Therefore, our results indicate that sinusoidal thrombosis may not be essential to induce hepatic fibrosis in the setting of elevated central venous pressure. We also realized that the degree of liver fibrosis varies in PAB rats regardless of similar sinusoidal dilatation, indicating that other factors in addition to congestion must contribute to hepatic fibrosis in CH. A low CO causes relative tissue hypoxia in all organs. The hepatic arterial buffer response (HABR), in which liver blood flow increases when portal blood flow decreases, has been reported in the liver; however, portal blood does not increase when liver blood flow decreases. Thus, relative ischemia occurs in the liver easier than in other organs [16]. Although intrahepatic ischemia has been demonstrated in an alcoholic liver disorder model rat by immunostaining using pimonidazole [17], it has not yet been confirmed whether intrahepatic ischemia is observed in CH. To our knowledge, this is the first report demonstrating that hypoxia was mainly present in Zone 3 (the region around the central vein), where relative ischemia readily occurs, in the PAB group (Fig 5). Accordingly, HIF-1α mRNA expression tended to be higher, and VEGF mRNA level was significantly increased in the liver of PAB rats (Fig 6). Consistent with this, the expression level of HIF-1α protein was significantly higher in the liver of PAB rats (Fig 9). In addition, HIF-1α was significantly localized in the nucleus of the PAB liver, suggesting the activation of HIF-1α. It has already been reported that hepatic fibrosis is related with hypoxia [11–13]. Moon et al. found that hepatic fibrosis was reduced in a HIF-1α knockout mouse after induction of hepatic cirrhosis by bile duct ligation [18]. HIF-1α knockout mice showed a decrease in mRNA expression of profibrotic mediators such as PDGF, fibroblast growth factor-2 (FGF-2), and plasminogen activator inhibitor-1(PAI-1). It is known well that hypxia induces PDGF, FGF-2, and PAI-1 [19–21] and that HIF-1α affects PAI-1 in hypoxic hepatocytes *in vitro* [20]. These studies support our hypothesis that low CO promoted hepatic fibrosis through tissue hypoxia in PAB rats.

Regarding the evidence of liver fibrosis, in addition to the histological changes, we detected a significant increase in the expression level of the fibrosis-promoting factors, such as TGF-β1 and CTGF, in the liver of PAB rats. Furthermore, we also found that the expression levels of MMPs and TIMPs mRNAs were increased in the liver of PAB rats. It has been known that the expression levels of MMP2, MMP9, TIMP1 and TIMP2 mRNAs are increased in the patients with hepatic fibrosis [22]. Recently, Chunqiu et al. reported that CTGF activate hepatic stellate cells through MMP2 and MMP9 [23]. Moreover, we also found that hyaluronic acid, a biomarker of hepatic fibrosis, was increased in serum of PAB rats. Taken together, these results indicate that PAB promoted hepatic fibrosis in the present study.

### PAB caused RV pressure overload and right heart failure

A limitation of the present study is that we did not directly measure CO and the pressure of central vein and the RV. However, in our unpublished data using different PAB rats, CO measured by echocardiography was strongly correlated to that measured by cardiac catheterization (S1 Fig). Therefore, we think that the data of CO are reliable in the present study. Regarding the relation between central venous pressure and liver fibrosis, in another unpublished data using different rats, RV pressure by cardiac-catheterization significantly correlated with the blood flow velocity in the PAB region on echocardiography in PAB rats (n = 29) (S2 Fig). Therefore, we estimate that the blood flow velocity measured by echocardiography could be an alternate to RV pressure in the present study. It should be noted that CO was maintained in some rats, but decreased in others; however, RV pressure was similarly high in both groups. We thought that the initial intensity of stenosis may have been stronger in animals with low CO and that the pressure gradient decreased with the development of RV failure.

In our study, RA dilatation was noted in animals with a decreased CO in the PAB group (S2 Fig). Since a relationship has been reported between central venous pressure and RA volume [24], we suggest that central venous pressure could be higher in rats with more right atrial dilatation. Therefore, RA dilatation and reduced CO may represent right heart failure in the decompensated stage. On autopsy, a large volume of ascites had accumulated in PAB rats with low CO, and nutmeg-like liver congestion was macroscopically observed (Fig 1B).

In addition to the limitation described above, we could not directly measure hepatic blood flow such as hepatic artery, hepatic vein, and portal vein. Thus, the degree of the decrease in hepatic blood flow by PAB still remains unclear. However, it is widely accepted that CO correlates with the tissue blood flow. Furthermore, due to HABR, portal blood flow does not increase even if liver blood flow decreases. Therefore, relative ischemia easily occurs in the liver more than in other organs.

### Clinical implications

The present study emphasizes that liver fibrosis needs to be accounted for a complication of right heart failure. Right heart failure is commonly problematic in patients with adult congenital heart diseases, especially after the Fontan procedure or surgery for the tetralogy of Fallot [25, 26]. Lindway et al. reported that hepatic cirrhosis was found in 55% of patients after Fontan procedure [27]. Camposilvan et al. demonstrated that liver dysfunction correlated with decreasing CO in patients after Fontan procedure [28], suggesting a strong relationship between reductions in CO and hepatic fibrosis.

Idiopathic pulmonary arterial hypertension also causes right heart failure. However, to our knowledge, there are few reports investigating liver fibrosis in patients with pulmonary arterial hypertension. Takeda et al. found that elevation of serum bilirubin is a risk factor for death in patients with pulmonary arterial hypertension, although they did not examine the prevalence of liver fibrosis [29]. It should be noted that significant liver damage, judged by routine blood chemically examination, was not obvious in PAB rats, even though they developed liver fibrosis. Our results suggest that there are more patients with RV pressure overload who develop latent liver fibrosis. Hyaluronic acid is known to be a useful marker of hepatic fibrosis [30], which is consistent with the present study. Although it has not been a routine blood chemically examination in right heart failure, our study suggests that examining hyaluronic acid is necessary to evaluate hepatic fibrosis in patients with right heart failure unless routine blood chemically examinations are normal. To date, it remains unclear whether developing liver fibrosis affects liver function and further cardiac dysfunction, and whether or not liver fibrosis is reversible. Further clinical and experimental investigations are required to address these issues.

## Conclusion

A PAB rat model was appropriate for evaluating CH. Hepatic fibrosis in CH advanced as a result of the progression of heart failure with reductions in CO. Tissue hypoxia was observed in the liver and HIF-1α expression was enhanced in animals with decompensated-stage heart failure, in which cardiac output decreased, suggesting that tissue hypoxia contributes to hepatic fibrosis.

## Supporting Information

S1 Fig Correlation between CO measured by echocardiography and that measured by catheter examination. RV: right ventricular; RA: right atrium; PA: pulmonary artery; CO: cardiac output. Red circle plot: pulmonary artery banding model rats (n = 5); Blue square plot: sham-operated controls (n = 5).
(TIF)

S2 Fig Correlation between the cardiac parameters measured by echocardiography and those measured by catheter examination. RV: right ventricular; RA: right atrium; PA: pulmonary artery. Red circle plot: pulmonary artery banding model rats (n = 29); Blue square plot: sham-operated controls (n = 10). The RV pressure was measured using a catheter. RV-PA pressure gradient, RA dimension and cardiac output were calculated using echocardiography.
(TIF)
