# Supplementary figures and images for: Low Cardiac Output Leads Hepatic Fibrosis in Right Heart Failure Model Rats
Source: PLoS One. 2016 Feb 10;11(2):e0148666. doi: 10.1371/journal.pone.0148666 (PMC4749189; doi:10.1371/journal.pone.0148666)

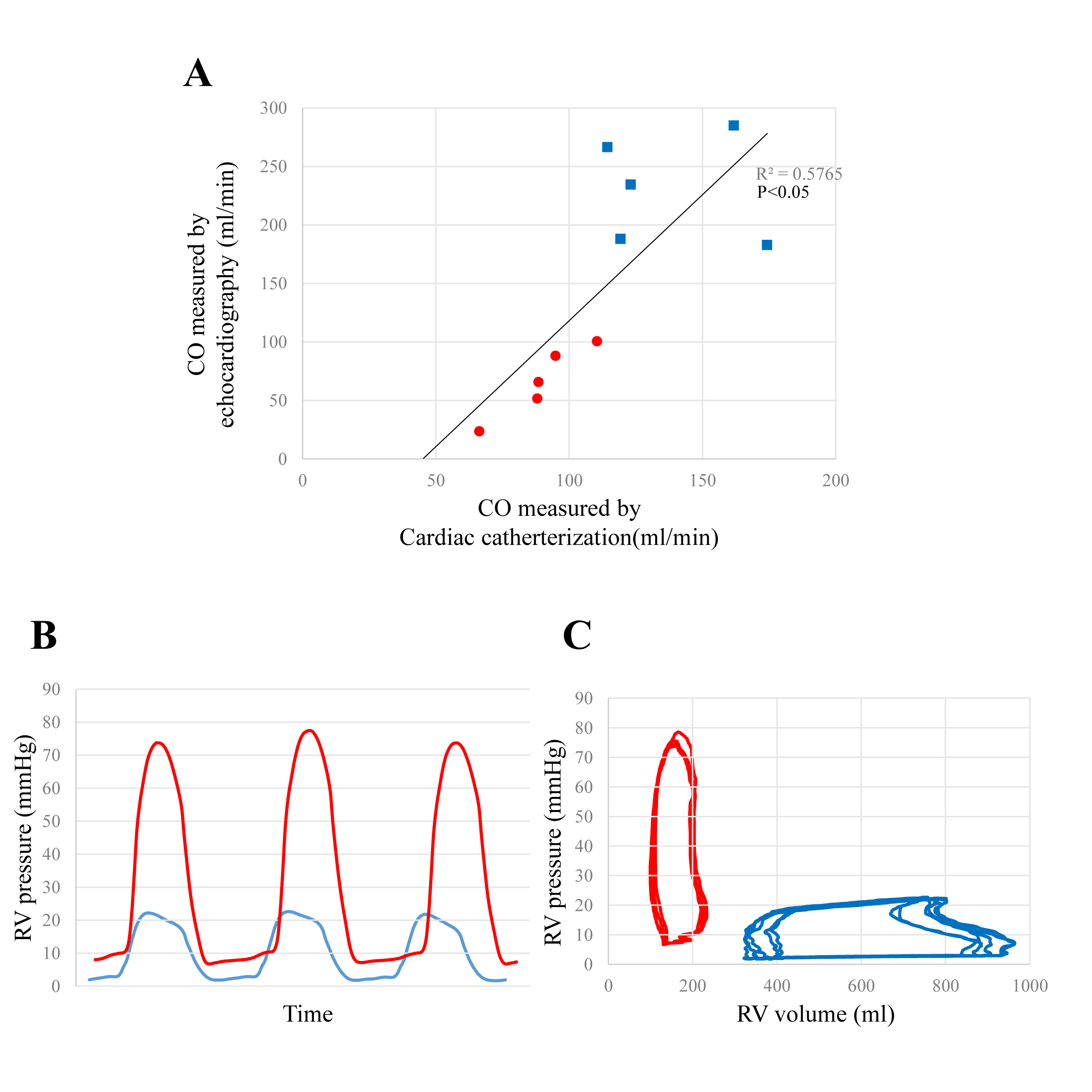

Supplement: S1 Fig — RV: right ventricular; RA: right atrium; PA: pulmonary artery; CO: cardiac output. Red circle plot: pulmonary artery banding model rats (n = 5); Blue square plot: sham-operated controls (n = 5). (TIF) [file pone.0148666.s001.tif]

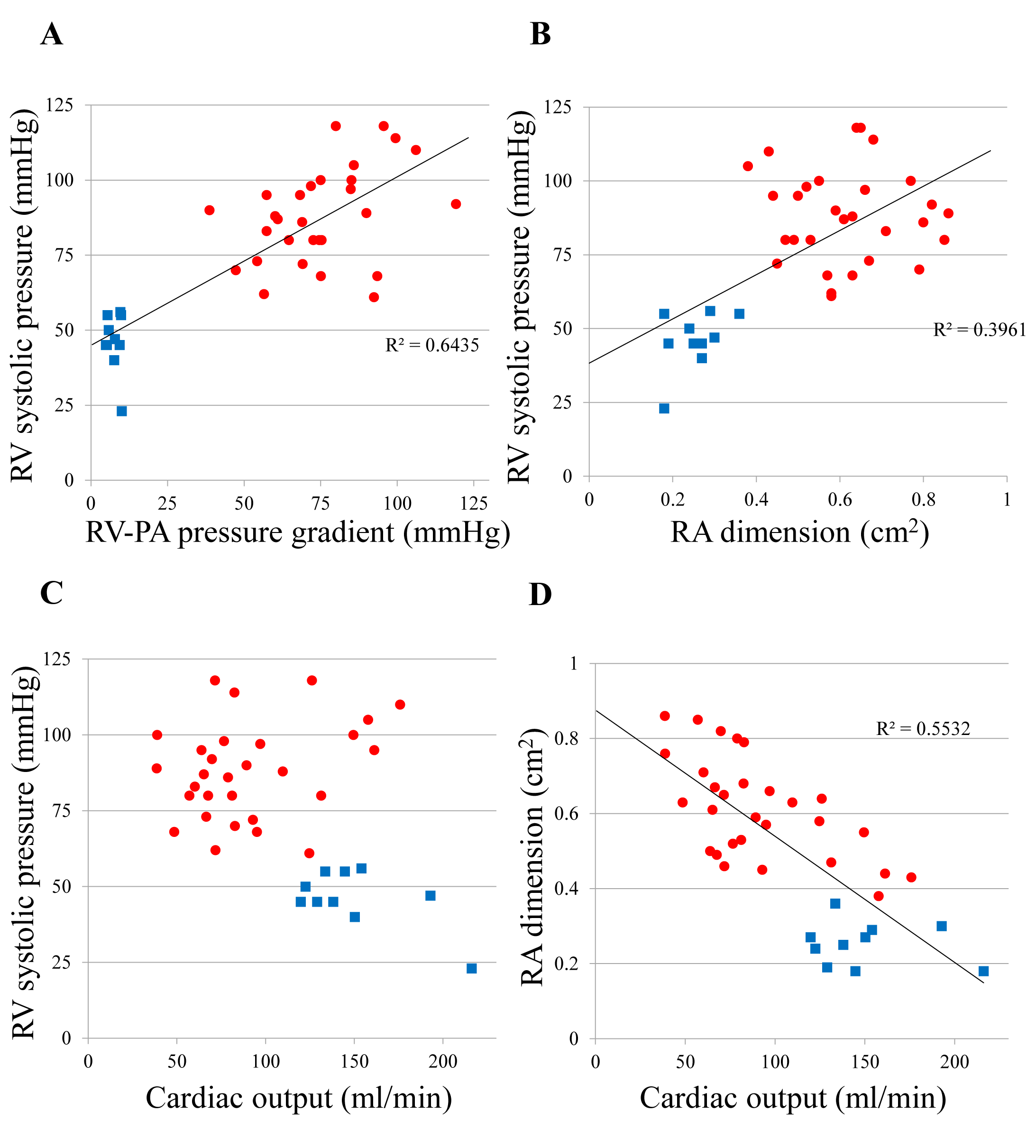

Supplement: S2 Fig — RV: right ventricular; RA: right atrium; PA: pulmonary artery. Red circle plot: pulmonary artery banding model rats (n = 29); Blue square plot: sham-operated controls (n = 10). The RV pressure was measured using a catheter. RV-PA pressure gradient, RA dimension and cardiac output were calculated using echocardiography. (TIF) [file pone.0148666.s002.tif]
